# A presumed pathological complete response of ruptured hepatocellular carcinoma showing retained intratumoral blood flow after trans-arterial chemo-embolization

**DOI:** 10.1016/j.radcr.2023.08.042

**Published:** 2023-08-24

**Authors:** Hiroshi Shintani, Shoji Oura

**Affiliations:** Department of Surgery, Kishiwada Tokushukai Hospital, Kishiwada-city, Japan

**Keywords:** Hepatocellular carcinoma, Intra-abdominal bleeding, Pathological complete response, Retained intratumoral blood flow, Trans-arterial chemo-embolization

## Abstract

A 74-year-old women with abdominal pain emergently visited our hospital in a shock status. After hemodynamics stabilization with intravenous fluid/albumin administration and blood transfusion, image evaluation showed perihepatic presumed blood retention and an intrahepatic large tumor. Angiography showed a tumor stain in the liver and no active leakage of the contrast medium from the tumor. These findings led to the diagnosis of ruptured hepatocellular carcinoma (HCC) without active bleeding. The patient, therefore, was treated not with trans-arterial embolization (TAE) but with trans-arterial chemo-embolization (TACE) using 10 mg of epirubicin. Post-TACE images showed marked tumor shrinkage with retained intratumoral blood flow. Under the tentative diagnosis of shrunken but viable HCC, the patient underwent laparoscopic segmentectomy for the HCC. Postoperative pathological study showed coagulative and lytic necrosis, intratumoral bleeding, hemosiderin deposits, massive collagen fiber, infiltration of inflammatory cells, and no viable cancer cells in the resected tumor. These pathological findings highly suggested that chemotherapeutic effect of epirubicin had brought about complete cancer cell death in the area not affected by TAE. Physicians should treat the patients with ruptured HCC, especially when showing stable hemodynamics, not by TAE but by TACE for better clinical outcome. Oncologists should further note that a complete pathological response of HCC could be observed even in cases of retained intratumoral blood flow.

## Introduction

For patients with oncologically operable hepatocellular carcinoma (HCC), surgery can provide superior relapse-free survival to any other therapeutic modalities. Many HCC patients, however, have no choice but to functionally choose embolization therapy due to their underlying diseases such as hepatitis [1,2] and liver cirrhosis [3]. Compared to other solid malignancies, overwhelmingly high local recurrence rate further makes physicians to treat HCC patients not with surgery but with embolization.

HCCs are generally hyper vascular tumors and, especially when exposing themselves directly to the abdominal cavity, sometimes cause intra-abdominal hemorrhage, often making physicians to emergently treat the ruptured HCC. Even in this situation, physicians are faced with 2 therapeutic options, that is, surgery and embolization, to control bleeding.

Response evaluation criteria in solid tumor (RECIST) [4] is commonly used to assess the therapeutic efficacy against solid malignancies. It, however, is not uncommon especially for large HCCs to show complete pathological responses even if tumor shadows remain after treatment. Modified RECIST (mRECIST) [5], therefore, has come to be used to correctly judge the therapeutic effect on HCC. mRECIST determines that a pathological complete response of the target tumor is obtained when blood flow within the shrunken tumor disappears completely.

We herein report a case of HCC showing a presumed pathological complete response with retained intratumoral blood flow after trans-arterial chemo-embolization (TACE) [6].

## Case report

**Fig. 1:**
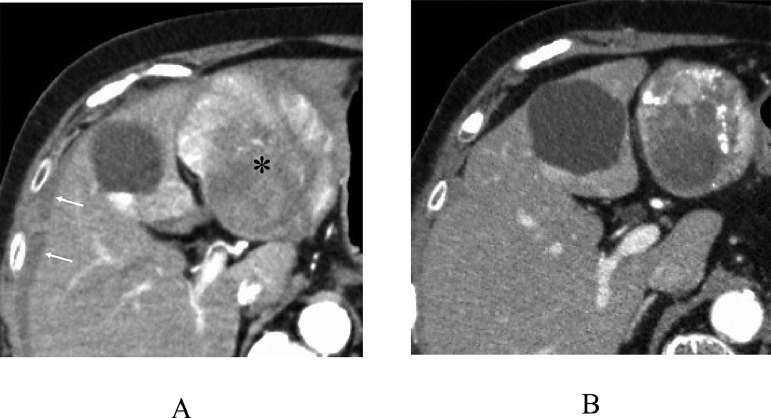
Enhanced computed tomography (CT) of the liver. (A) Enhanced CT just after stabilization of the patient's hemodynamics showed an oval mass, 83 mm in size (asterisk), with heterogenous enhancement and small amount of perihepatic fluid (arrows). (B) Enhanced CT 5 weeks after trans-arterial chemo-embolization showed an oval mass, 61 mm in size, with intratumoral blood flow.

**Fig. 2:**
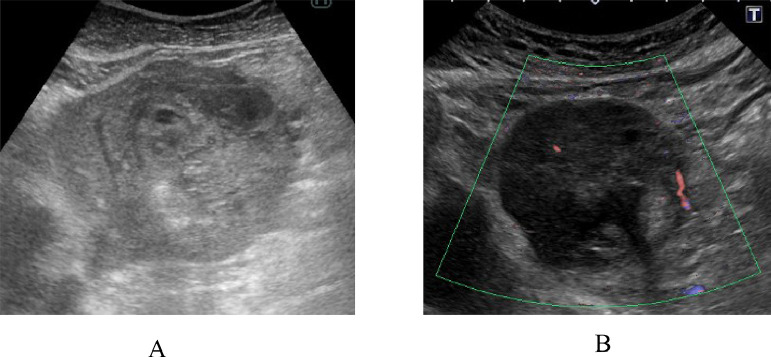
Ultrasonography of the hepatocellular carcinoma (HCC) after trans-arterial chemo-embolization (TACE). (A) Ultrasonography 6 days after TACE showed an oval mass with relatively high internal echoes. (B) Ultrasonography 48 days after TACE showed marked reduction of the tumor size, conversion of the internal echoes from high to low, and intratumoral blood flow.

**Fig. 3:**
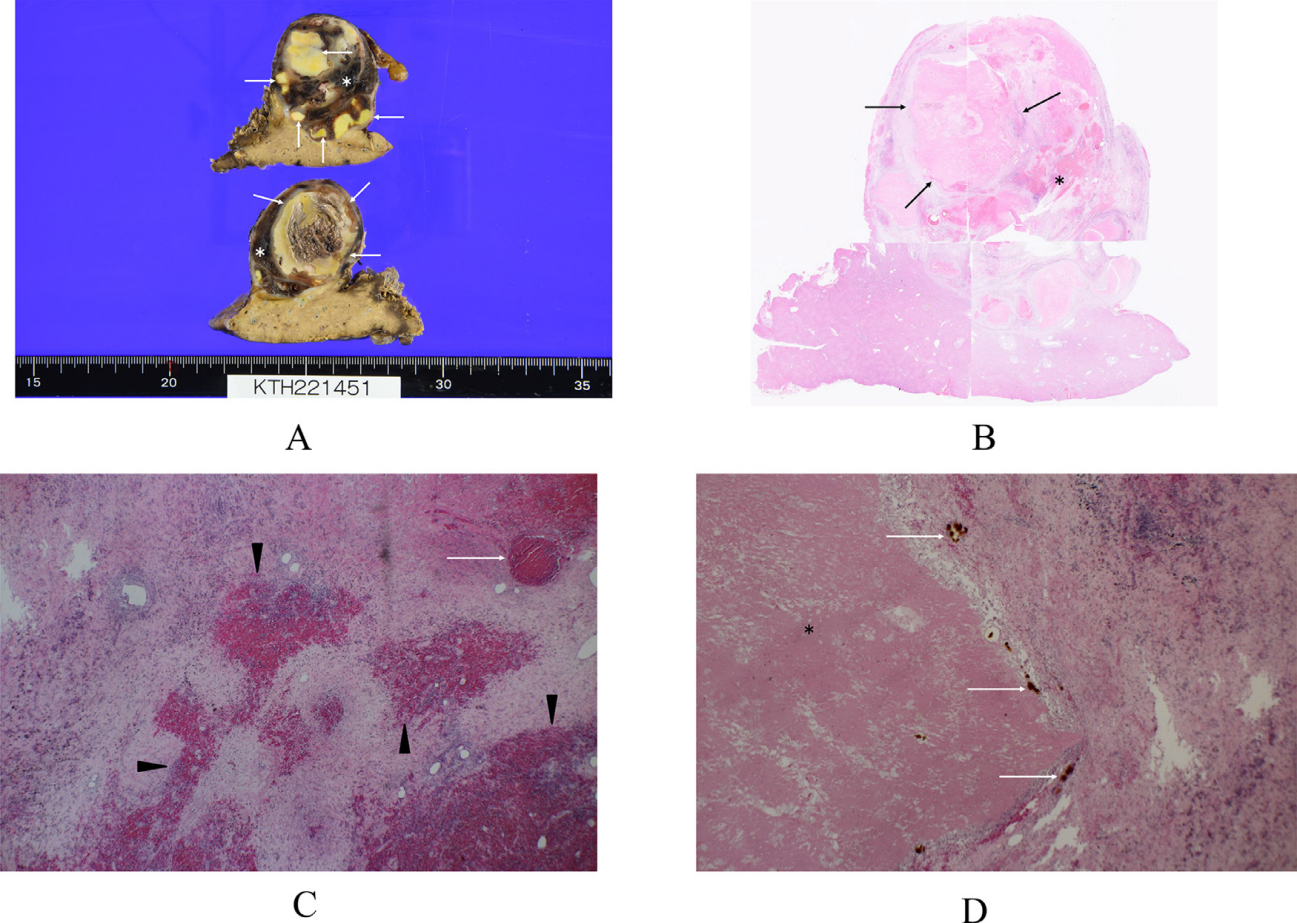
Postoperative pathological findings. (A) Macroscopic findings showed an oval tumor, 36 mm in size, in which a cream-colored area (arrows) indicated the coagulative and lytic necrosis due to embolization and a brown-colored area located between the coagulative/lytic area and the fibrous capsule (asterisks). (B) Low-magnified view of the tumor showed an area of coagulative and lytic necrosis (arrows) and intratumoral bleedings (asterisk). (C) Magnified view showed a presumed micro-vessel (arrow) and intratumoral hemorrhage (arrowheads). (D) Magnified view showed hemosiderin deposits (arrows) adjacent to the coagulative necrosis area (asterisk).

A 74-year-old women without hepatitis B and C infection visited our hospital for emergent treatment of her abdominal, that is., from epigastrium to the upper right abdomen, pain. On her arrival to our hospital, the patient was in a shock status with systolic blood pressure of around 50 mmHg. The patient, therefore, received intravenous fluid/albumin administration and blood transfusion, leading to the stabilization of her hemodynamics. Taken in parallel with these treatment, enhanced computed tomography (CT) showed an oval mass, 83 mm in size, in the liver segment 3 with early enhancement and a washout pattern (Fig. 1A) and small amount of perihepatic presumed blood. Selective angiography to the liver lesion, begun in only 98 minutes after her arrival to our hospital, showed a tumor stain without overt extrahepatic leakage of the contrast-medium. These findings suggested that the tumor had ruptured with a certain amount of bleeding into the abdomen but had no active bleeding at the time of angiography. The patient, therefore, was treated not with trans-arterial embolization (TAE) [7] but with TACE using 10 mg of epirubicin. TACE required 62 minutes of catheter intervention procedures but did not re-deteriorate the once-stabilized hemodynamics of the patient. The patient recovered uneventfully and was discharged on the 10th day after TACE. Ultrasonography showed an oval mass with relatively high internal echoes on the 6th day followed by tumor shrinkage both with low internal echoes and retained intratumoral blood flow on the 48th day after TACE (Fig. 2). Enhanced CT (Fig. 1B), taken 5 weeks after TACE, also showed intratumoral early enhancement followed by a typical washout pattern, strongly suggesting the presence of residual viable tumor cells. The patient, therefore, underwent laparoscopic segmentectomy, in approximately 2 months after TACE, for the HCC under the preoperative assessment of clinically resectable but at least partially viable HCC. Macroscopic findings of the bisected formalin-fixed tumor clearly showed an oval tumor, 36 mm in size, with obviously demarcated 2 areas (Fig. 3A). Postoperative pathological study showed one area consisting of massive coagulative and lytic necrosis and the other area consisting of intratumoral bleeding, hemosiderin deposits mainly just adjacent to the coagulative necrosis area, massive collagen fiber, infiltration of inflammatory cells, and no viable cancer cells (Figs. 3B-D). After the operation, the patient had an uneventful course, was discharged from the hospital on the 15th postoperative day, and has been well for 6 months without any HCC recurrences.

## Discussion

Unlike other organs, dual blood supply, that is, hepatic artery and portal vein, to the liver allows physicians to treat HCC with TAE or TACE. TAE is a feasible liver-directed therapy for nonsurgical candidates not eligible for local thermal ablation. TACE, however, has been performed much more frequently than TAE due to its higher antitumor efficacy against HCC. Therefore, Barcelona Clinic Liver Cancer group [8,9] includes the TACE but not TAE in the treatment algorithm of HCC.

On active hemorrhage of HCCs, physicians have 2 major therapeutic options such as open surgery or catheter intervention. Which should be selected is determined by patient's condition, hardware characteristics of the medical facility, and doctor's skill. In centers where emergency angiography is available, catheter hemostasis is generally preferred over open surgery for its less invasiveness. Of the 2 embolization modalities, TAE is more often chosen because the primary goal of treatment in this situation is not oncological cure but hemostasis. However, if selective hepatic angiography after the onset of HCC rupture shows little or no active bleeding with stable patient's hemodynamics, TACE may be a feasible therapeutic option even in this setting.

We performed pathological evaluation not with multiple sections, but with a maximal section in this case. We, therefore, cannot exclude the possibility of minute viable HCC residuals in the tumor. Macroscopic and microscopic findings, however, showed that the coagulative and lytic necrosis area caused by embolization effect did not contain any viable cancer cells. In addition, the area between the fibrous capsule and the coagulative and lytic necrosis area was fully occupied by fibrous tissue, lymphocytes, and bleeding at least on the maximal cut surface. These pathological findings, therefore, should highly suggest that the addition of epirubicin chemotherapy brought about a complete pathological response to this area.

Hemoglobin changes over time from oxyhemoglobin into deoxyhemoglobin, intracellular methemoglobin, and extracellular methemoglobin, and is finally converted to hemosiderin by being phagocytized by macrophages. Conversion from oxyhemoglobin to extracellular methemoglobin needs approximately 2 weeks. Therefore, hemosiderin deposits observed just adjacent to the coagulative necrosis area highly suggest that not embolization effect but epirubicin chemotherapy effect should have brought about complete tumor cell death in the noncoagulative necrosis area in this case.

A complete response by RECIST means complete disappearance of the target tumor(s). Complete tumor disappearance can occur only after total tumor cell death by some kind of antitumor treatment followed by subsequent phagocytosis by immune cells. for example, macrophages. Therefore, the smaller the tumor, the easier it is to totally phagocytize the nonviable tumor cells. Conversely, if a tumor is large, the tumor shadow remains even if a pathological complete response is obtained. HCCs are often found as large tumors with fibrous capsules. Since the fibrous capsule itself does not generally have tumor cells, it often remains after some kind of anti-HCC treatment even if achieving total cancer cell death. mRECIST was proposed in 2010 to compensate for the shortcomings of RECIST. In this amended criteria, complete disappearance of intratumoral arterial enhancement in all target lesions was also defined as a complete response. In this case, hemosiderin deposits, bleeding, inflammatory cell infiltrates, fibrous tissue, and no viable HCC cells were observed outside the coagulative necrosis area in the shrunken tumor. Hemosiderin deposits suggest the phagocytosis of extracellular methemoglobin by macrophages, implying the incomplete embolization of the HCC. These findings well explain the biphasic pathological findings by arterial embolization effect and the chemotherapy effect. In other words, antitumor effects observed between the fibrous capsule and the coagulative/lytic necrosis area were definitely caused by the epirubicin chemotherapy effect.

## Conclusion

In this case, why intratumoral blood flow existed even after achieving a complete pathological response remains uncertain. Oncologists, however, should select TACE for hemodynamically stable patients with ruptured HCCs and note that a pathological complete response can be observed in HCC patients with retained intratumoral blood flow.
